# Supplementary material for: AMPK signaling to acetyl-CoA carboxylase is required for fasting- and cold-induced appetite but not thermogenesis
Source: eLife. 2018 Feb 13;7:e32656. doi: 10.7554/eLife.32656 (PMC5811211; doi:10.7554/eLife.32656)
Supplement: Figure 2—figure supplement 1—source data 2. [file elife-32656-fig2-figsupp1-data2.zip › Figure 2 - supplement 1 - source data 2.pptx]

## Slide 1
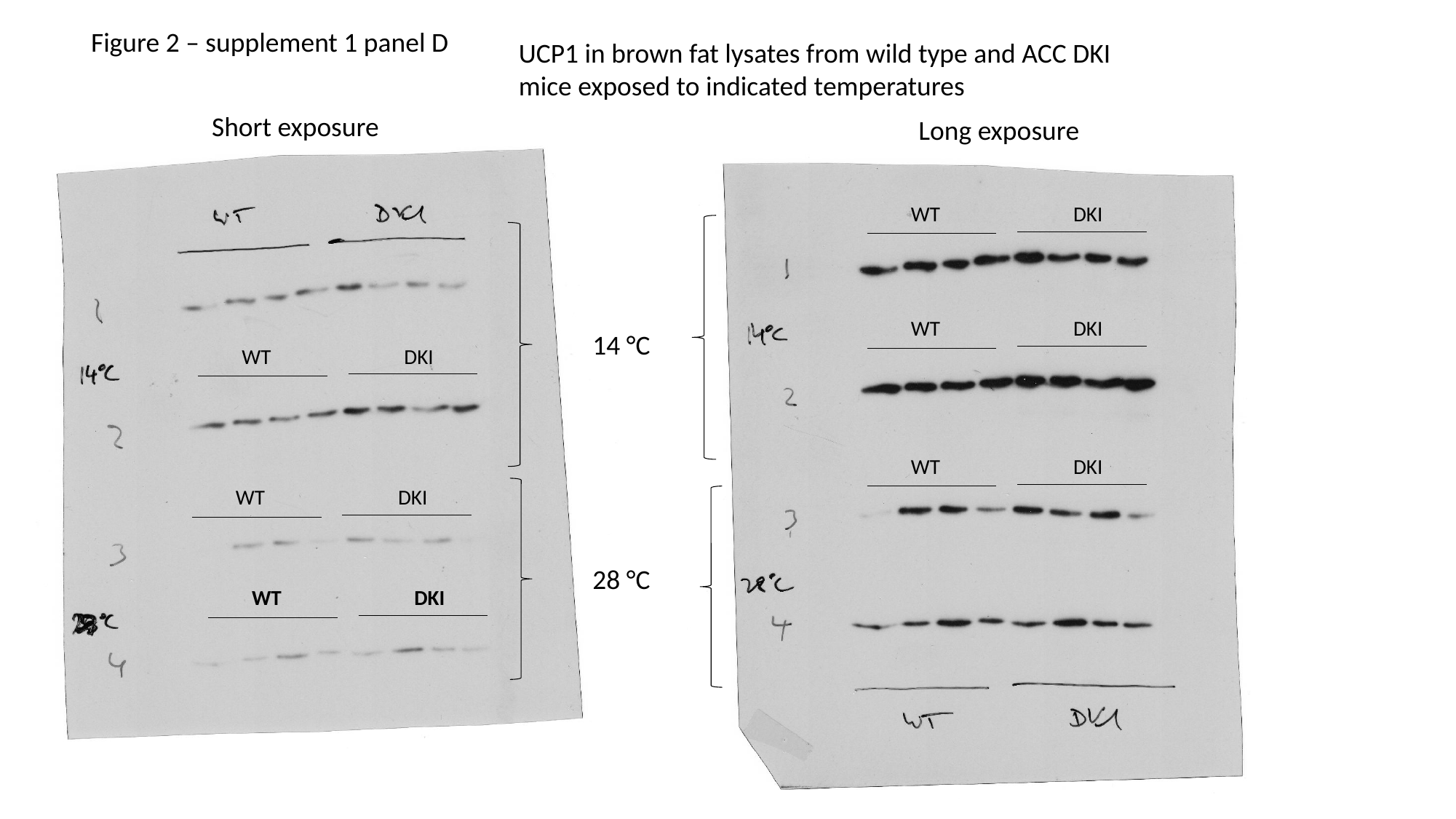

Figure 2 – supplement 1 panel D
UCP1 in brown fat lysates from wild type and ACC DKI mice exposed to indicated temperatures
Short exposure
Long exposure
WT
DKI
WT
DKI
14 °C
WT
DKI
WT
DKI
WT
DKI
28 °C
WT
DKI

## Slide 2
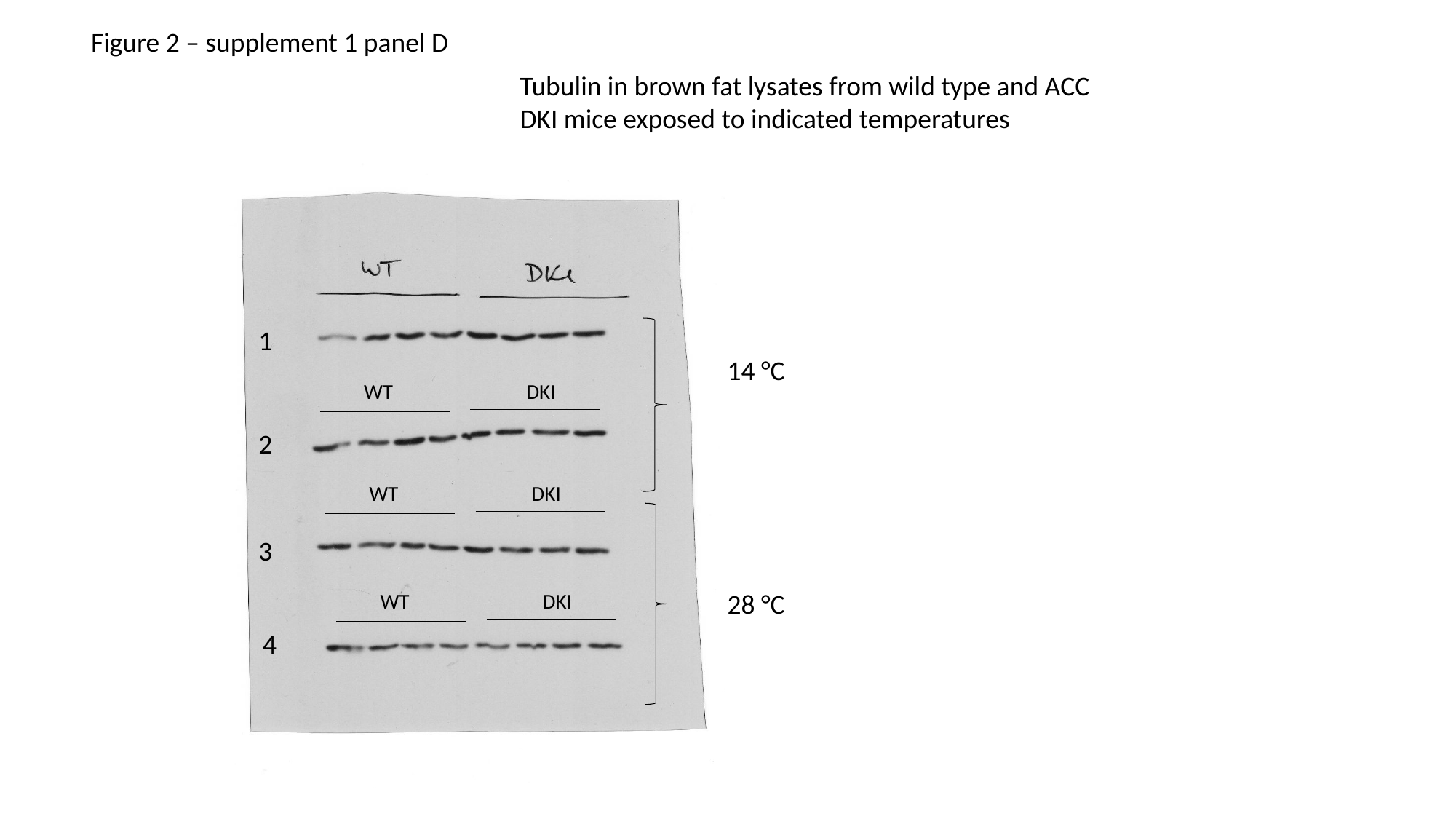

Figure 2 – supplement 1 panel D
Tubulin in brown fat lysates from wild type and ACC DKI mice exposed to indicated temperatures
1
14 °C
WT
DKI
2
WT
DKI
3
28 °C
WT
DKI
4
