# Supplementary material for: AMPK signaling to acetyl-CoA carboxylase is required for fasting- and cold-induced appetite but not thermogenesis
Source: eLife. 2018 Feb 13;7:e32656. doi: 10.7554/eLife.32656 (PMC5811211; doi:10.7554/eLife.32656)
Supplement: Figure 3—figure supplement 1—source data 1. [file elife-32656-fig3-figsupp1-data1.zip › Figure 3 - supplement 1 - source data 1.pptx]

## Slide 1
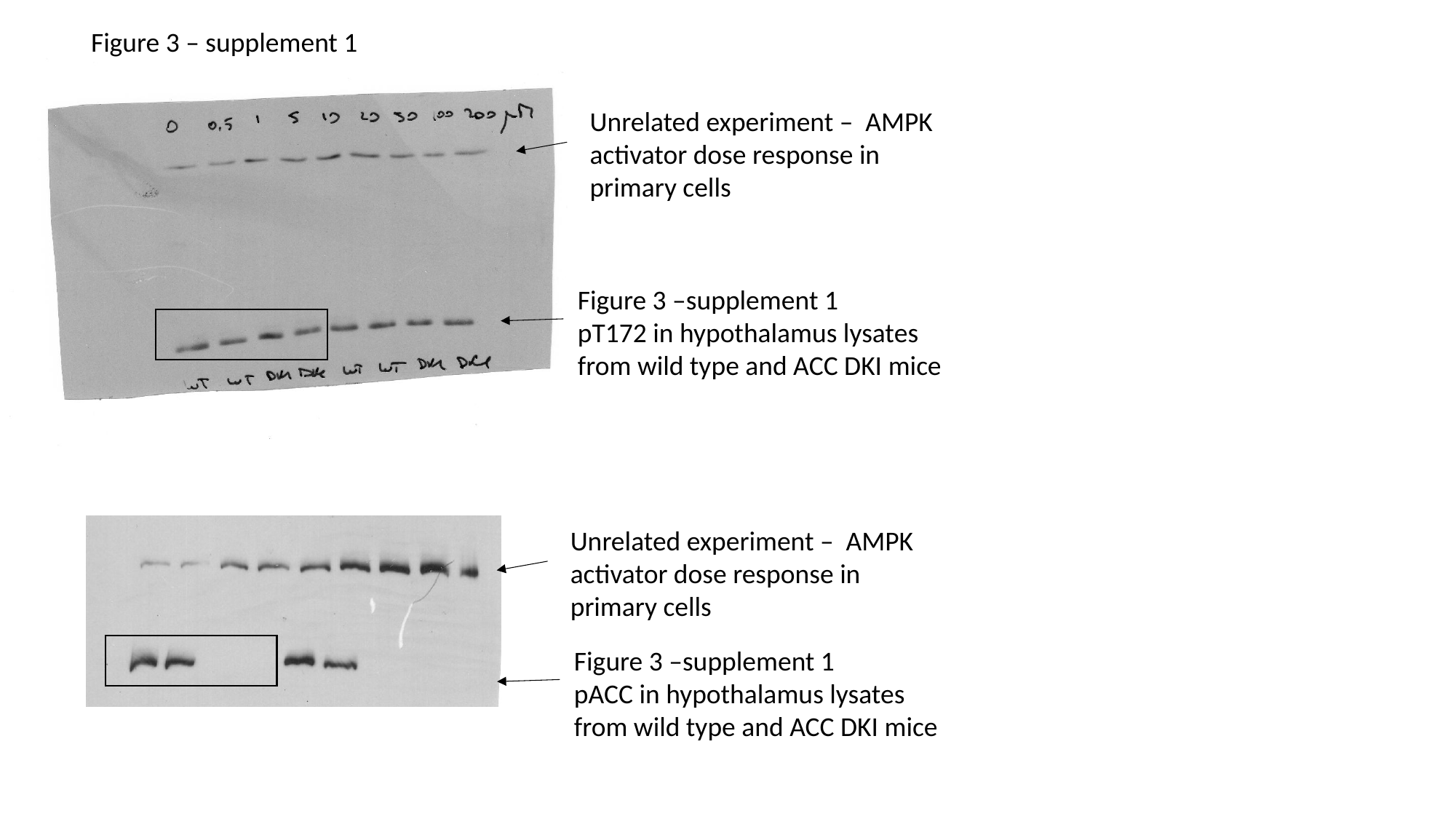

Figure 3 – supplement 1
Unrelated experiment – AMPK activator dose response in primary cells
Figure 3 –supplement 1
pT172 in hypothalamus lysates from wild type and ACC DKI mice
Unrelated experiment – AMPK activator dose response in primary cells
Figure 3 –supplement 1
pACC in hypothalamus lysates from wild type and ACC DKI mice

## Slide 2
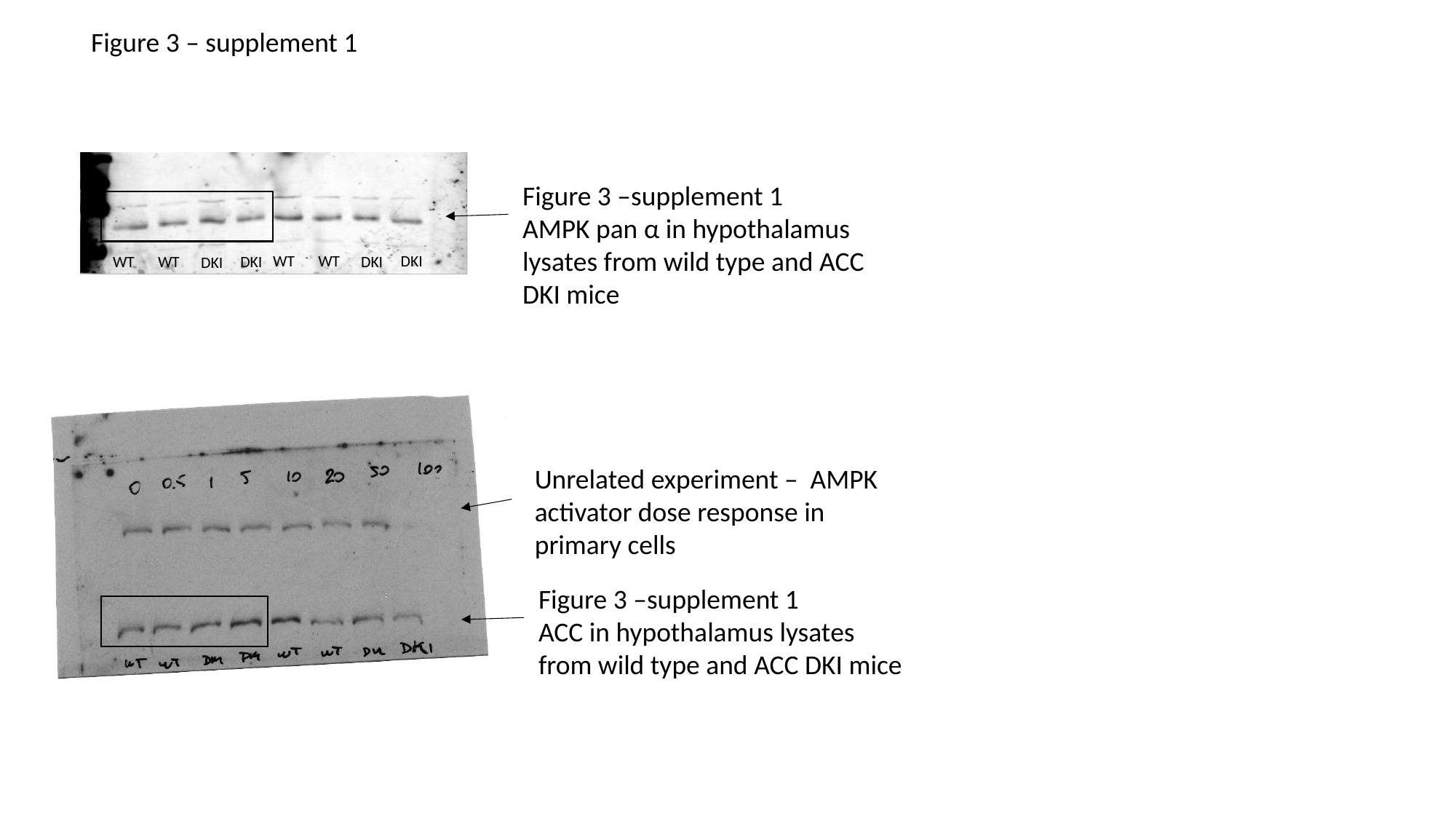

Figure 3 – supplement 1
Figure 3 –supplement 1
AMPK pan α in hypothalamus lysates from wild type and ACC DKI mice
WT
WT
DKI
WT
DKI
WT
DKI
DKI
Unrelated experiment – AMPK activator dose response in primary cells
Figure 3 –supplement 1
ACC in hypothalamus lysates from wild type and ACC DKI mice

## Slide 3
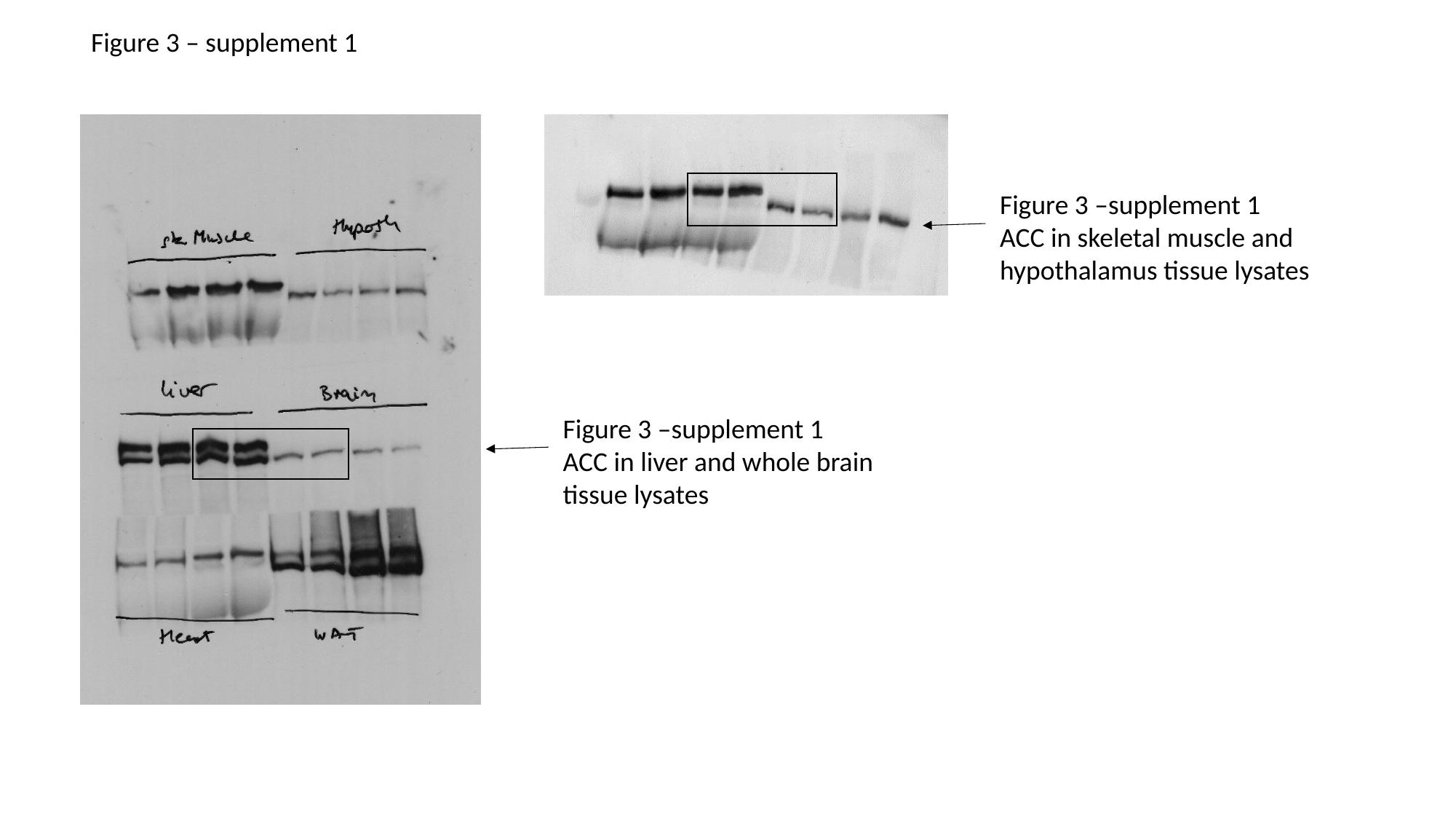

Figure 3 – supplement 1
Figure 3 –supplement 1
ACC in skeletal muscle and hypothalamus tissue lysates
Figure 3 –supplement 1
ACC in liver and whole brain tissue lysates
