# Supplementary material for: AMPK signaling to acetyl-CoA carboxylase is required for fasting- and cold-induced appetite but not thermogenesis
Source: eLife. 2018 Feb 13;7:e32656. doi: 10.7554/eLife.32656 (PMC5811211; doi:10.7554/eLife.32656)
Supplement: Figure 6—source data 2. [file elife-32656-fig6-data2.zip › Figure 6 - source data 2.pptx]

## Slide 1
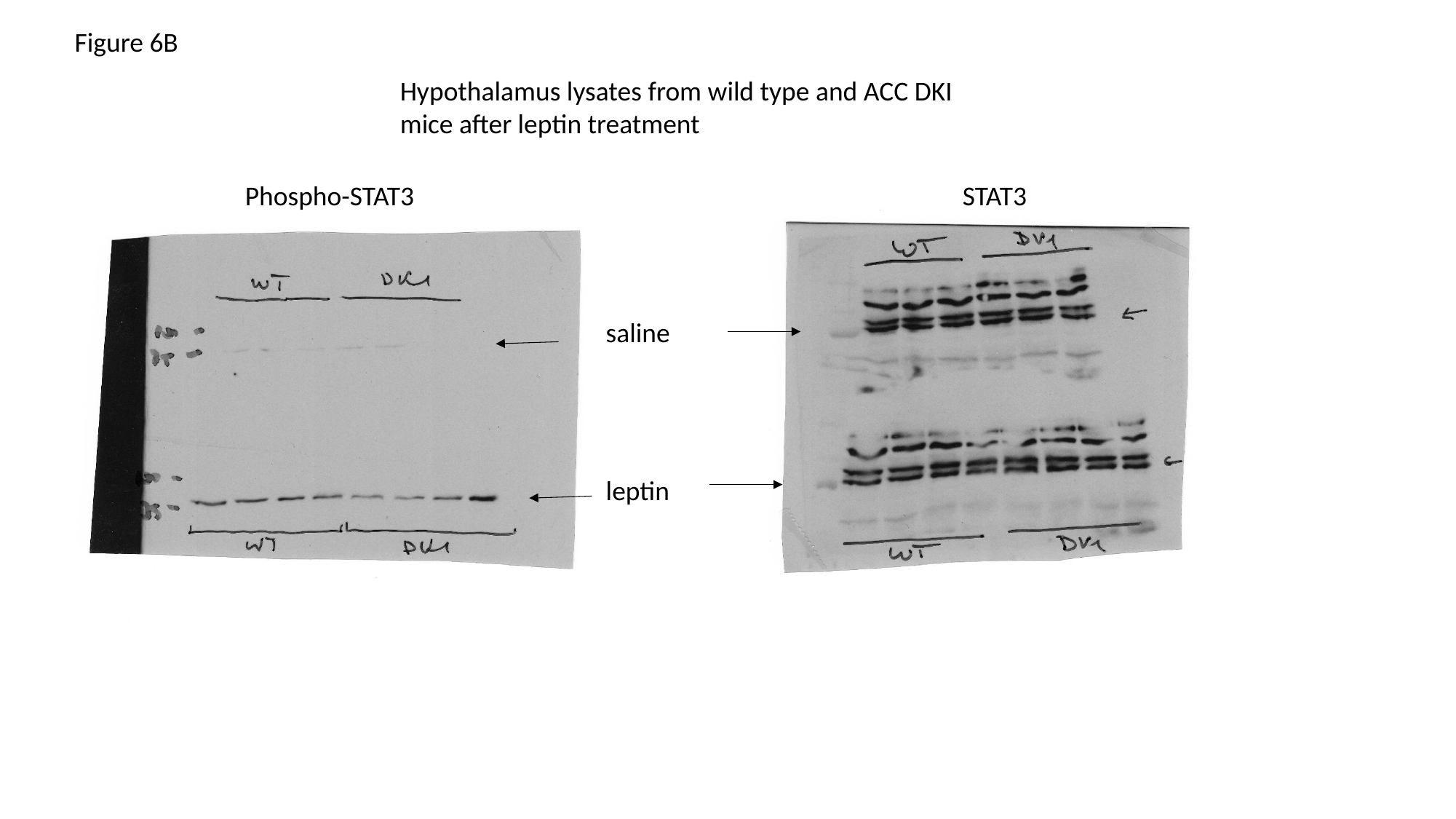

Figure 6B
Hypothalamus lysates from wild type and ACC DKI mice after leptin treatment
Phospho-STAT3
STAT3
saline
leptin
