# Supplementary material for: AMPK signaling to acetyl-CoA carboxylase is required for fasting- and cold-induced appetite but not thermogenesis
Source: eLife. 2018 Feb 13;7:e32656. doi: 10.7554/eLife.32656 (PMC5811211; doi:10.7554/eLife.32656)
Supplement: Supplementary file 1. — Assays were purchased from Applied Biosystems and consist of a pair of unlabeled PCR primers and a TaqMan probe with a FAM dye label. [file elife-32656-supp1.docx]

| **Gene symbol** | **Gene name** | **TaqMan Assay** |
| --- | --- | --- |
| 18S | Eukaryotic 18S rRNA | Hs99999901_s1 |
| *Acaca* | Acetyl-CoA carboxylase 1 | Mm01304257_m1 |
| *Acacb* | Acetyl-CoA carboxylase 2 | Mm01204671_m1 |
| *Acadm* | Medium chain acyl-CoA dehydrogenase | Mm00431611_m1 |
| *Acadl* | Long chain acyl-CoA dehydrogenase | Mm00599660_m1 |
| *Agrp* | Agouti related neuropeptide | Mm00475829_g1 |
| *Cartpt* | Cocaine and amphetamine regulated transcript | Mm04210469_m1 |
| *Cd36* | CD36 antigen | Mm00432403_m1 |
| *Cidea* | Cell death-inducing DNA fragmentation factor, alpha subunit-like effector A | Mm00432554_m1 |
| *Cpt1b* | Carnitine palmitoyl transferase 1b | Mm00487191_g1 |
| *Elovl3* | Elongation of very long chain fatty acids protein 3 | Mm00468164_m1 |
| *Fasn* | Fatty acid synthase | Mm0062319_m1 |
| *Npy* | Neuropeptide Y | Mm01410146_m1 |
| *Pomc* | Pro-opiomelanocortin α | Mm00435874_m1 |
| *Ppara* | Peroxisome proliferator activated receptor α | Mm00440939_m1 |
| *Ppargc1a* | Peroxisome proliferative activated receptor, gamma, coactivator 1 α | Mm00447183_m1 |
| *Scd1* | Stearoyl-Coenzyme A desaturase 1 | Mm00772290_m1 |
| *Srebf1* | Sterol regulatory element binding transcription factor 1 | Mm00550338_m1 |
| *Ucp1* | Uncoupling protein 1 | Mm01244861_m1 |

**Table S1. List of TaqMan Gene Expression Assays used for qRT-PCR**.

Assays were purchased from Applied Biosystems and consist of a pair of unlabeled PCR primers and a TaqMan probe with a FAM dye label.
